# Peeling Plaids Apart: Context Counteracts Cross-Orientation Contrast Masking

**DOI:** 10.1371/journal.pone.0008123

**Published:** 2009-12-02

**Authors:** Elliot Freeman, Preeti Verghese

**Affiliations:** 1 Department of Psychology, City University, London, United Kingdom; 2 Smith Kettlewell Eye Research Institute, San Francisco, California, United States of America; University of New South Wales, Australia

## Abstract

**Background:**

Contrast discrimination for an image is usually harder if another image is superimposed on top. We asked whether such contrast masking may be enhanced or relieved depending on cues promoting integration of both images as a single pattern, versus segmentation into two independent components.

**Methodology & Principal Findings:**

Contrast discrimination thresholds for a foveal test grating were sharply elevated in the presence of a perfectly overlapping orthogonally-oriented mask grating. However thresholds returned to the unmasked baseline when a surround grating was added, having the same orientation and phase of either the test or mask grating. Both such masking and ‘unmasking’ effects were much stronger for moving than static stimuli.

**Conclusions & Significance:**

Our results suggest that common-fate motion reinforces the perception of a single coherent plaid pattern, while the surround helps to identify each component independently, thus peeling the plaid apart again. These results challenge current models of early vision, suggesting that higher-level surface organization influences contrast encoding, determining whether the contrast of a grating may be recovered independently from that of its mask.

## Introduction

Discrimination of fine changes in the contrast of a target stimulus is often impaired in the presence of a superimposed overlay mask, at suprathreshold contrasts [1]–[4]. This masking effect is weaker from non-overlapping stimuli surrounding the target [3], [5], though interestingly, surrounds begin to affect contrast thresholds when the target edges merge with a same-orientation and same-phase surround [6]. Such findings suggest that masking interactions depend on scene segregation mechanisms, such that surround interactions are reduced when target and surround may be identified as discrete regions [6], [7]. This therefore raises the question of how the local contrast response in early vision is related to the perceptual appearance and global interpretation of the stimulus, as modulated by cues for segmentation. We address this here for the case of ‘plaids’ [8]–[10].

Plaids offer a convenient stimulus for investigating the role of integration mechanisms in building complex two-dimensional patterns out of simple one-dimensional components. When drifting gratings cohere to form a plaid, observers perceive a checkerboard composed of bright and dark elliptical blobs, drifting in a unique direction relative to each of the components [10]. It can then become very difficult to individuate the properties of the components. For example, observers cannot judge the speed of one grating independently of the other under conditions where plaid coherence is perceived, but interestingly, speed discrimination recovers under conditions that promote a segmented percept [11], [12]. Here, we tested whether an analogous plaid segmentation effect may also be observed in the contrast domain. It is well known that contrast discrimination of a static test grating is impaired in the presence of a superimposed orthogonally-oriented grating [1]–[3], [13]–[16]. For drifting gratings, is such contrast masking from the orthogonal component abolished under conditions of plaid segmentation?

We investigate this question here using a new contextual manipulation that can readily induce perceptual segmentation of a plaid into its constituent components. Two orthogonal drifting gratings that appear to cohere into a plaid when they overlap perfectly, may be perceptually segmented when the boundaries of one component are extended beyond the boundaries of the other. We first present subjective data confirming the efficacy of this manipulation (Experiment 1). We then measure contrast discrimination thresholds under conditions that correspond to perceptual coherence and perceptual segmentation (Experiment 2 and 4) to test the hypothesis that segmentation counteracts the masking effect found when the grating components cohere. To relate our studies to the known effects of contrast masking on static gratings, we measure increment thresholds on a static version of the drifting stimuli (Experiment 3) and compare these data to other masking studies on static gratings as well as to our own data on drifting gratings (Experiment 2 & 4). Finally, in Experiment 5 we controlled for a possible explanation of masking and unmasking based on surround suppression.

## Results

### Experiment 1: Perceived Drift Direction

When two orthogonally oriented gratings with otherwise identical characteristics are superimposed, the resulting plaid pattern typically appears to drift along a vector at 45° to the two component vectors, with its direction and velocity determined by the intersection-of-constraints principle [10]. It is well known that when the two drifting components are the same size, they can cohere strongly. This experiment examined whether coherence is reduced when one of the components (designated the “mask”), is twice the size of the test. Coherence was assessed from the perceived drift direction of the central pattern.

**Figure 1:**
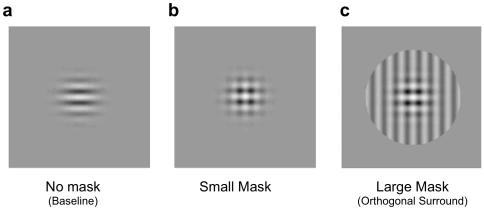
Examples of stimuli. a) horizontal Test grating; b) plus superimposed vertical Small Mask grating with same dimensions as Test; c) plus same vertical mask, but now extended into surround.

Observers indicated the perceived direction of a central Test grating drifting at random cardinal or oblique drift directions on successive trials, in the presence of an overlapping orthogonally-oriented Mask of the same size or of double the diameter (see Figure 1 and Supplementary Information files ‘Audio/Video S1’ and ‘Audio/Video S2’ respectively, for movies of typical examples of Small Mask and Large Mask stimuli). Responses for each trial were counted as indicating ‘Plaid’ motion if the response was in the intersection-of-constraints direction, for example up and to the left when the two components drifted upwards and leftwards respectively. In the Large Mask condition, a response was counted as moving in the ‘Component’ direction if the observer reported the actual direction of the central small grating. For the Small Mask condition, a response in either the direction of the Test or the Mask grating was classified as ‘Component’, as there was no cue indicating which component in particular was the test. A third category counted responses in neither component nor plaid directions. The number of Plaid or Component responses was then expressed as proportions of the total number of responses (which also included the third category).

**Figure 2:**
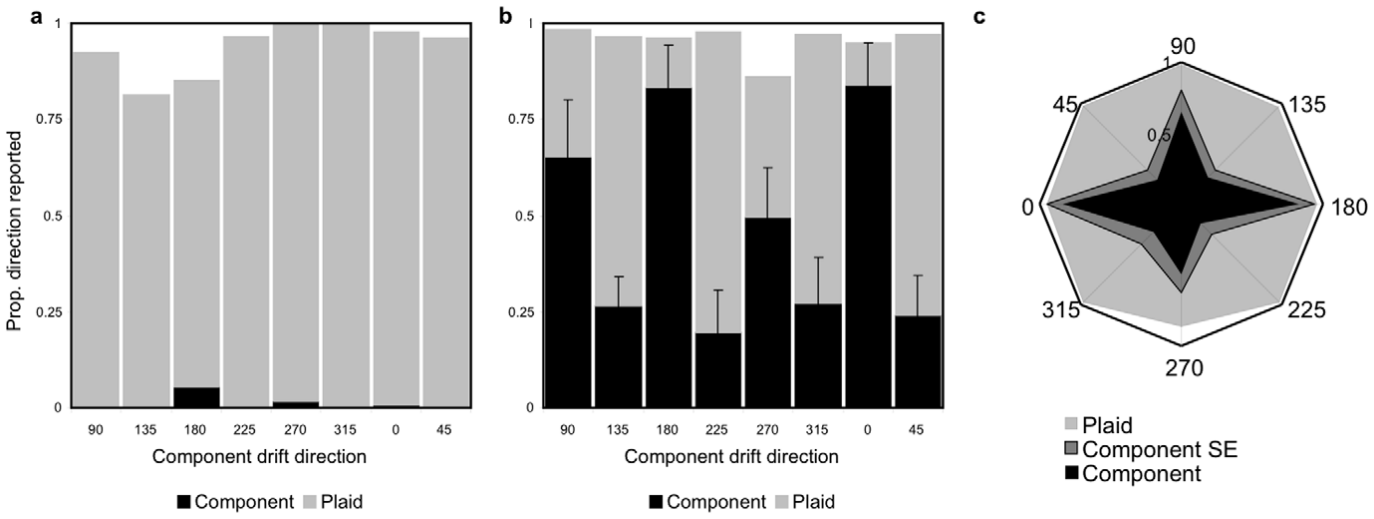
Results from Experiment 1, summarized across six observers. a) Small Mask condition. Stacked bar chart represents the proportion of trials in which motion was reported in the component direction (black, with errorbars representing one unit of Standard Error), in the plaid direction (gray), and all other directions (white). b) Large Mask condition, showing proportional increase in component motion reports. c) Radial plot displaying the proportion of component and plaid motion reports by actual component drift direction, for the Large Mask condition only. Mid gray sections represent one unit of Standard Error.

Results are displayed as stacked barcharts for each Test stimulus direction, with within-subjects standard error bars (Figure 2b). The relative proportion of trials in which motion was reported in the component and plaid directions is plotted as a function of test direction. Figure 2a and 2b plot perceived direction in the Small and Large Mask cases respectively. Figure 2c replots the results for the Large Mask condition on a radial graph, with the proportion of component responses shown towards the centre of each graph (plus within-subjects standard-error margins), and the remaining proportion of plaid responses shown on the outside. It is clear from these graphs that when component motion was reported, it was almost exclusively for the Large Mask condition. An ANOVA comparing the proportion of component responses (relative to all possible responses) confirmed a significant difference between Large and Small Mask conditions [F(1,7) = 25.75, *p* = .001], as well as a significant main effect of test drift orientation [F(4,28) = 11.27, *p*<.0001] and two-way orientation by mask size interaction [F(4,28) = 12.14, *p*<.0001]. For the Large Mask condition only, the proportion of component motion reports varied significantly as a function of the orientation of the central test stimulus, with greater segmentation observed for cardinal as opposed to oblique drift directions (see Figure 2c) [F(7,28) = 6.04, *p*<.0002]; no significant anisotropy was found for the Small Mask condition [F(7,28) = 1.58, ns].

These results support our initial hypothesis that the contextual manipulation of the plaid surround induces perceptual segmentation of the plaid components, thus allowing the motion direction of the Target component to be individuated. The specific preference for motion in cardinal directions concurs with prior evidence of the classical oblique effect for direction discrimination [17], [18], where sensitivity to motion direction is higher in cardinal than in oblique directions.

### Experiment 2: Contrast Discrimination of Moving Gratings

Our second experiment investigated whether the same contextual conditions that induced perceptual segmentation of plaid components in Experiment 1 also reduced the contrast masking effect of one component on the other. We compared contrast discrimination for a Test grating, either alone (‘Baseline’, Figure 1a), or in the presence of a superimposed orthogonally-oriented ‘Small’ or ‘Large’ Mask (Figs. 1b & 1c, respectively). The latter condition effectively added a surround annulus to the ‘small’ Mask, having the same orientation and phase as the central mask.

We used a two-interval forced choice discrimination paradigm in which the observer had to detect an increment in the contrast of a test grating (relative to a baseline or ‘Pedestal’ contrast of 30%). In the presence of a mask we predicted thresholds to be higher when the test coheres with the mask as in the Small Mask condition, and lower when the test is more likely to segment from the mask as in the Large Mask condition. Recall that Experiment 1 suggests that the small and large mask conditions promote subjective integration and segmentation, respectively of the test grating and the mask.

**Figure 3:**
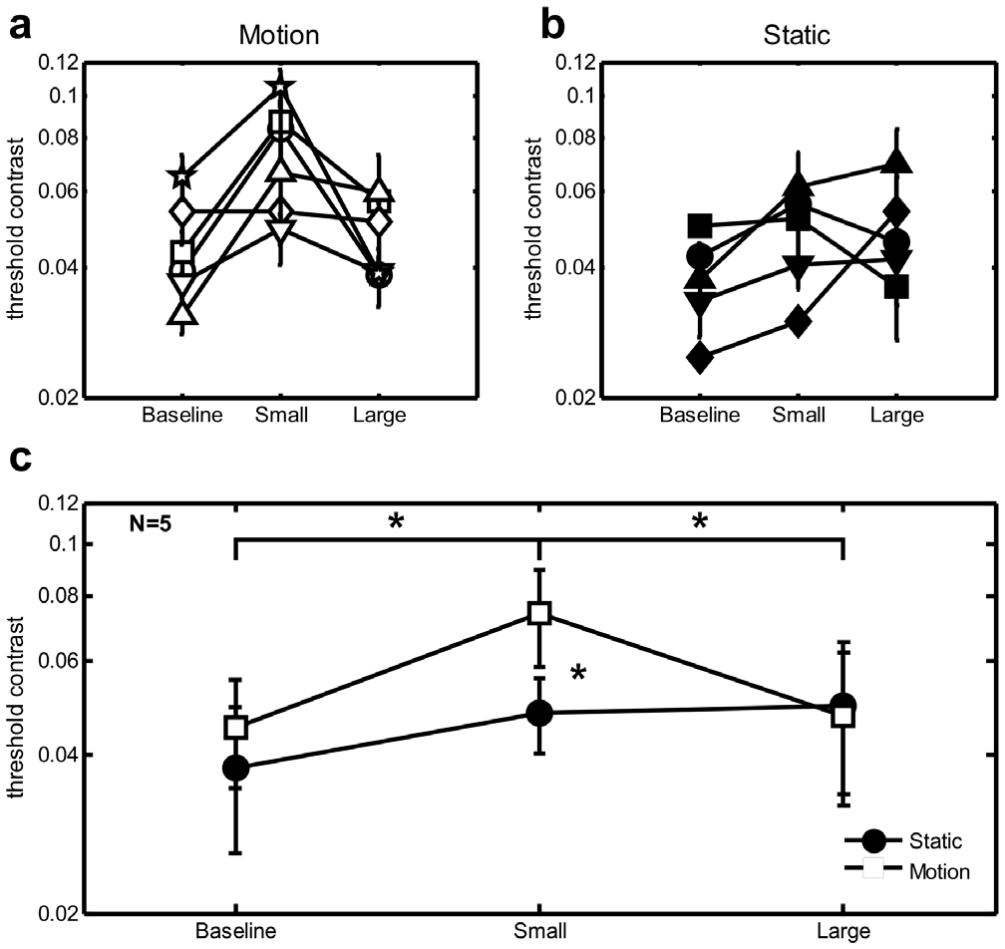
Results from Experiments 2 and 3. Contrast thresholds of individual observers, for small and large mask conditions relative to no-mask baseline, for (a) motion, and (b) static displays. Results are plotted in units of Michelson contrast on a log scale. Errorbars indicate one unit of Standard Error of the threshold estimate. c) Group means for Experiments 1 and 2. Filled and open symbols are for static versus motion displays respectively, with asterisks and brackets indicating significant differences between pairs of conditions at *p*<.05. Errorbars indicate 95% confidence intervals based on within-subjects Standard Error.

Contrast discrimination thresholds were estimated by fitting the psychometric functions with a Weibull curve, to find the increment contrast at which each observer was at 82% correct (see Figure 3a). In the Small Mask condition, thresholds were elevated by 70% on average (Standard Error 21%) relative to baseline [t(5) = 3.83, *p*<.02]. However in line with our ‘plaid-segmentation’ hypothesis, thresholds in the Large Mask condition dropped [paired-sample t(5) = 2.62, *p*<.05, relative to Small Mask] to levels not significantly higher than the no-mask baseline [13% increase, SE 20%, t(5) = 0.3, ns]. Thus, it appears that in the Large Mask condition, segmentation of the test and mask counteracts the masking effect (see Figure 3a).

### Experiment 3: Contrast Discrimination of Static Gratings

Drifting plaid stimuli have most often been used to examine motion integration [10], while static ‘plaid’ stimuli composed of orthogonal components have traditionally been used to examine the mechanisms of contrast gain control [1]–[3], [13]–[16]. None to our knowledge have measured contrast discrimination for moving plaids, as we did in Expt. 2, nor directly compared this with static plaids, which we did in this experiment. With five of the original six participants of Expt. 2 we performed a replication to test whether the masking and unmasking effects might generalize to static instead of drifting stimuli. Methods were identical to Expt. 2, except that all stimuli were presented without drifting motion.

Masking effects were considerably weaker with static stimuli compared to the drifting stimuli tested in Experiment 2. The Small Mask produced only 28% (SE 11%) threshold elevation with respect to the baseline condition, of borderline significance [t(4) = 2.67, *p* = .056]. The Large Mask produced a slightly larger elevation on average (42% relative to baseline, SE 30%), though thresholds were neither significantly different from the Small Mask [t(4) = .21, ns] nor baseline conditions [t(4) = 1.38, ns] (see Figure 3b). This replicates past findings [15] that increment thresholds are little affected by mask size for static stimuli.

A two-way repeated-measures ANOVA, across the five observers who served in both motion and static experiments, confirmed that the mask size manipulation had significantly different effects under moving versus static conditions [F(1,4) = 10.00, *p*<.05], with no significant main effects (see Figure 3c, plotting mean thresholds with within-subjects 95% confidence intervals). Post-hoc comparisons revealed that only the thresholds with the small mask were significantly higher in motion compared to static [t(4) = 3.48, *p* = .013].

The above comparison of Experiments 2 and 3 provides new evidence that contrast overlay masking, though historically assessed using static stimuli, may actually be considerably stronger in the presence of motion. Here we offer a tentative explanation based on perceptual integration versus segmentation. If decreased contrast discrimination thresholds with large masks are indeed associated with perceptual *segmentation* of Test and Large Mask components, as suggested from Expt. 2, then any elevation of thresholds, such as that previously observed with moving stimuli with small masks, might conversely result from enhanced perceptual *integration* of the components into a plaid pattern. The common motion direction of both components likely reinforces this integration. The smaller elevation of thresholds in the static Small Mask condition might be due to the weaker integration of the grating components into a plaid. The increased variability of the results with static stimuli suggests that the percept of a static plaid was more ambiguous than motion, with some observers tending to see integration more than others. If so, motion might act as a disambiguating cue, providing common-fate cues that serve to bias perception towards integration, at least in the absence of counteracting contextual cues. This interpretation remains speculative at present, as the method used successfully to assess subjective coherence or segmentation with moving plaids in Experiment 1 could not be used with static plaids.

### Experiment 4: Threshold Versus Contrast Functions for Moving Stimuli

An overlaying and/or surrounding mask can sometimes benefit instead of impair discriminability of the central test stimulus, depending on factors such as the relative orientation and spatial frequency of the relative to the target, or the contrast of the components [e.g. 15], [19], [20]. In the present case, it is therefore possible that the apparent benefit of the Large Mask found with moving stimuli might just be specific to the contrast that we tested, and that this benefit might be eliminated or even reversed at different test stimulus contrasts. In the contrast discrimination tasks so far (Experiments 2 & 3), we measured the difference threshold (i.e. the Just Noticeable Difference) for detecting a contrast increment on a fixed ‘pedestal’ contrast of 30%. In this experiment we replicated Experiment 2 across a wide range of pedestal contrasts, from subthreshold to 30%, to obtain a function relating difference threshold to the pedestal contrast (i.e. a ‘Threshold versus Contrast’ function or TvC). This approach has two advantages. Firstly we may establish to what extent the Small versus Large Mask effects observed with motion stimuli generalize across a range of contrasts or are merely accidental to just one contrast level. Secondly, the resulting TvC may be used to constrain a quantitative model that describes how the presence of the mask and its size might influence different types of non-linear interactions between early visual mechanisms.

**Figure 4:**
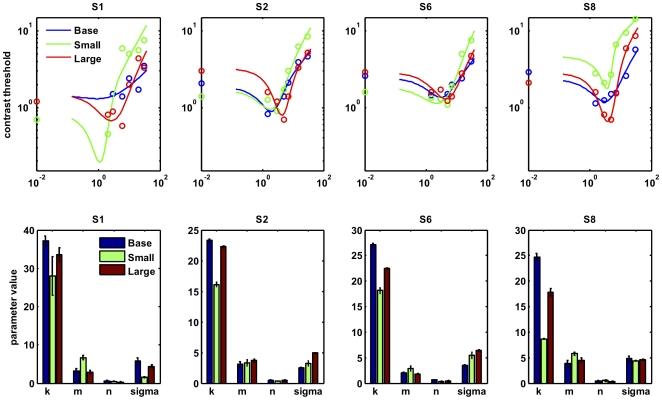
Experiment 4 results. Top row: Threshold versus Contrast data and best-fitting functions for four observers, on log-log axes. Bottom row: parameter estimates for the function fit, with bootstrapped 95% confidence intervals. See Equation (1) and main text for explanation.

Figure 4a shows the TvC functions for each observer, which have the classical ‘dipper’ shape [20]. In all observers thresholds are consistently higher for the Small Mask than for Large Mask and Baseline Conditions across a wide range of suprathreshold contrasts up to 30%, thus replicating the results obtained in Experiment 2. The benefit of the surround is therefore not an accidental finding restricted to our choice of a 30% contrast pedestal in Experiments 1 & 2 (addressing the first of our goals, above). Moreover it would be difficult to explain the reduction of this benefit with static stimuli by arguing that static stimuli merely had different effective contrasts compared to moving [21].

Concerning our second goal, the precise shape of the TvC can shed light on how the response to the target grows with contrast in the presence of different masks [20]. Here, we briefly review how the TvC relates to the non-linear response of the underlying contrast-sensitive mechanisms (i.e. the contrast transducer function) [2], [20], [see also illustration in ref. 22]. In the classical literature [2], [20], it is commonly assumed that the response initially follows an accelerating function of pedestal contrast, that saturates at higher pedestal contrasts. A Just Noticeable difference (JND) in contrast is generated when the response of this mechanism increases by a criterion value. As response is a non-linear function of pedestal contrast, the increment contrast required to generate a JND depends on pedestal contrast. For example, around absolute threshold (i.e. at zero pedestal contrast), only a small increment in contrast is required to yield a JND; however as pedestal contrast increases, and the corresponding transducer function continues to accelerate, progressively smaller increments are required. The smallest contrast increment (in the region of the dipper) is required at a pedestal contrast corresponding to the steepest part of transducer function. As the transducer function begins to decelerate beyond this point, further increases in pedestal contrast require progressively larger increments to generate a JND. From this point, contrast discrimination thresholds increase again, following Weber’s law.

Characteristics of an empirical dipper function, such as the depth of the dipper, the leftwards-rightwards or vertical position of the dipper, depend on the parameters of the transducer function which determine how steeply it accelerates, the strength of suppression at higher contrasts, and the overall gain. Here we consider a generic model of the contrast transducer function that includes such parameters [e.g. 23], [24]:
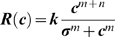
(1)


where the response R of a mechanism stimulated by the Target pedestal contrast ***c*** is subject to divisive normalization with an additive constant **σ**, before being amplified by an overall contrast-independent response gain parameter ***k***. The empirical TvC functions were fit using an algorithm based on ‘fminsearch’ in Matlab to obtain estimates of these parameters. It should be noted that this modelling was aimed primarily to capture the qualitative differences between fitted functions that differentially characterise a change in divisive inhibition or a change in response gain. Though better quantitative fits would likely be obtained with more sophisticated models including further parameters [e.g. 2], [16], the following qualitative features are common across models. Firstly, an increase in response gain parameter ***k*** (a constant by which the overall response of the mechanism is amplified) tends to translate the dipper function down the y-axis. Changes in spatial uncertainty are also known to translate the function vertically [e.g. 22], although the mechanism for this is not as simple as change in overall in response gain [25]. Secondly, an increase in divisive inhibition **σ** translates the entire transducer function rightward and thus drives the position of the minimum point of the ‘dipper’ rightwards along the x-axis. Finally, an increase in the exponent **m** controls the degree of the accelerating non-linearity, and thus makes the dipper more pronounced and thresholds rise more steeply at contrasts just above the dipper.

Figure 4 shows the resulting fits of the above model as curves superimposed on the empirical datapoints (top row), plus the parameter values for ***k***, ***m***, ***n***, and **σ** plotted as bar charts (bottom row). A consistent pattern is revealed across the four observers, where the response gain parameter ***k*** drops substantially with the Small Mask but recovers to baseline levels with the Large Mask. As ***k*** is associated with response gain, our data suggest a decrease in response gain that shifts the dipper upward for the small mask condition compared to the large mask condition. In addition, for three observers the Large Mask is associated with a small increase in the additive normalization parameter **σ** relative to the Small Mask, which accounts for the rightwards shift of the dipper in this condition. Confidence intervals (95%) for the parameter values were estimated via bootstrapping (100 samples), and are displayed in the bottom row of Figure 4 (bottom row) as error bars. For both **k** and **σ** parameters, non-overlapping confidence intervals indicate that the differences between conditions are significant at *p*<.05. The parameter **n** did not vary across conditions, while the parameter **m**, which makes the dipper more pronounced, is significantly elevated for the Small Mask condition in three observers.

These modelling results suggest that the small mask suppresses the overall response to the target (***k***), while adding the surround relieves this suppression; the surround also appears to slightly increase divisive inhibition (**σ**), with the result that sensitivity to the target is actually increased at mid-range contrasts.

### Experiment 5

Past psychophysical studies have shown that contrast sensitivity for a central grating may be suppressed by a ‘collinear’ annular surround with the same grating orientation and phase, but much less by an ‘orthogonal’ surround [1], [3], [5], [16], [26]–[28], with facilitation even found for the latter case [19], [29]. Such centre-surround interactions might explain the facilitation due to the large mask. For instance, target suppression from an overlaying small orthogonal mask might have been alleviated with a large orthogonal surround because this suppresses visibility of the central mask of the same orientation, thus improving target visibility.

**Figure 5:**
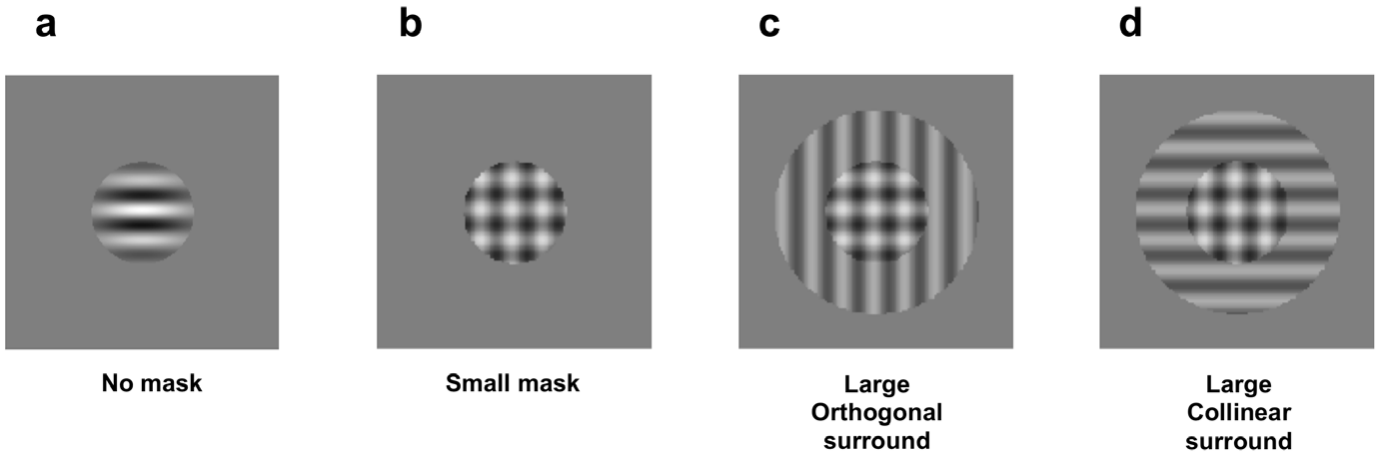
Stimuli for Experiment 5. a) Test stimulus plus pedestal alone; b) with superimposed small orthogonally-oriented mask; c) with ‘large’ orthogonal mask extended into surround; d) with a surround that is collinear relative to test stimulus.

To test this ‘surround-suppression’ hypothesis we tested contrast discrimination for a horizontal test grating (Fig. 5a) overlaid by a Small Mask (Fig. 5b), and adding either a vertical Orthogonal Surround (Fig. 5c, similar to the Large Mask in Expt. 1, Fig. 1c) or a new horizontal Collinear Surround (Fig. 5d). The latter was created by effectively rotating the original surround until it was no longer orthogonal with the test stimulus but now collinear (i.e. having the same phase and orientation). Only drifting stimuli were used, as in Expt. 2. Note that the target contrast increment still occurred within the same central area as before, and only this restricted area was masked by the now small orthogonal grating (Figure 5c). Note also that now the central region had sharp contrast boundaries in both Small and Large Mask conditions, unlike in Experiments 1–4 where the central region had a Gaussian edge (c.f. Fig. 5a–c with Fig. 1a–c). See Methods for further details.

If suppression from an iso-oriented surround reduces the effectiveness of the overlay mask in the original large mask condition, then the new Collinear Surround condition should result in enhanced masking, rather than reduced masking (as observed in the Large Mask condition in Expt. 1, and replicated here in the Orthogonal Surround condition). Indeed contrast discrimination thresholds should be higher than in the small-mask condition, as the Test stimulus should now be subject to even greater suppression from the additional collinear surround than from the small orthogonal mask by itself. In contrast, our plaid-segmentation hypothesis predicts the same unmasking effect regardless of the surround orientation.

**Figure 6:**
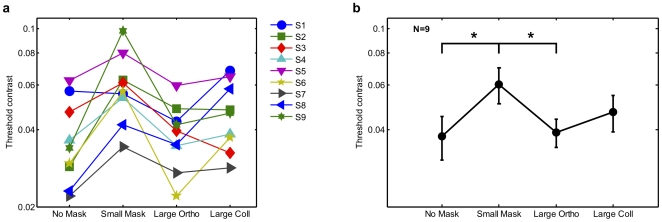
Experiment 5 results. a) Contrast thresholds for eight individual observers, plotted on a log scale, for the four stimulus conditions illustrated in Figure 5. b) Group means, with asterisks and brackets indicating significant differences between pairs of conditions, at p<.05.

Thresholds for this condition were obtained by both the Method of Constant Stimuli (MCS) and by an adaptive Quest procedure. All the observers except S5 and S7 participated in the Quest procedure. S5 & S7 and the two authors (S2 and S8) participated in the MCS procedure, (figure 6a). We fit psychometric data from both procedures with a Weibull function to facilitate a comparison of thresholds from these two methods. QUEST and MCS methods also provided very similar thresholds for the two observers who provided data for both (S2 & S8 in Figure 6a), and thus an average was taken across these to represent their final threshold estimate. Prior to statistical analysis we took the log of the thresholds and subtracted the baseline.

Thresholds are shown for each observer in Figure 6a and summarized as mean thresholds in Fig. 6b. A one-way ANOVA on mean thresholds relative to Baseline revealed a significant main effect of mask size/orientation [F(2,16) = 9.63, p<.002]. As before, thresholds for small-mask stimuli were elevated relative to baseline (71% elevation, SE 19%, one-way t(8) = 4.55, *p*<0.002). Thresholds were not significantly elevated for the Orthogonal surround mask (10% elevation, SE 11%, ns), nor for collinear surrounds (34%, SE 17%). Small mask thresholds were elevated significantly relative to Orthogonal surround [paired-sample t(8) = 4.66, *p*<0.002], and also elevated relative to the Collinear surround though not significantly [t(8 = 2.17, *p* = .06]. Collinear thresholds were slightly higher than Orthogonal masks but not significantly [t(8) = 2.11, *p = *.07].

The small threshold elevation observed for the Collinear surround relative to Orthogonal is partially consistent with some limited surround suppression from the Collinear surround to the central test stimulus. However if surround suppression were the whole explanation for the results observed so far, we should have observed thresholds that were significantly *higher* than in the Small mask condition, not lower as it appears here. Thus these results support the predictions of the plaid-segmentation hypothesis (in combination with some limited surround-suppression), whereby any surround that matches the orientation of one of the central plaid components helps to peel them apart.

## Discussion

Our experiments demonstrate that subjective visibility and contrast discrimination of a test stimulus can both be modulated by contextual surround cues that reinforce perceptual segmentation from (versus integration with) a superimposed mask. We show firstly that the perceived direction of motion switches from the compound (intersection of constraints) direction to the component direction in the presence of a surround. We further find that the same contextual manipulation improves contrast discrimination for a test component, apparently relieving masking from the cross-oriented overlay. This masking and unmasking effect is considerably stronger for motion relative to static stimuli (Experiment 3).

Both the subjective perceived direction data and the objective contrast increment data are consistent with integration and segmentation effects. The results of the static versus motion comparison suggest that the common perceived motion direction of both grating components helped the gratings cohere into a plaid more effectively than in the static case; however for the large mask, the non-overlapping surround provided a cue for segmentation, thus effectively helping to peel the plaid apart again into its constituent components.

In past studies, improvements in contrast sensitivity have been observed as a result of changes in spatial attention [30], feature-based attention [31], or due to a reduction of spatial or feature uncertainty [25], [32]–[34]. Could any such factors explain the present results? For example, the addition of a surround might have induced a distribution of spatial attention over a larger area, thus diluting the resources available for the central discrimination. Alternatively, the surrounding context might have masked the boundaries of the central target area, thus increasing spatial uncertainty for the target's location. These accounts are unlikely given that the target area was always clearly demarcated, by the presence of the high-contrast central pedestal in Experiments 1–3 (and in Expt. 4 for suprathreshold pedestals), or by a sharp contrast step and markers in Experiment 5. The addition of these latter cues made no appreciable difference to the pattern of results. Furthermore, the above spatial attention and spatial uncertainty accounts would both predict higher contrast thresholds in the presence of the surround, contrary to what we found.

Feature-based attention [31] might have enhanced sensitivity for a central target sharing the same orientation as the surrounding context. Such feature-based attentional enhancement might in principle explain the benefits observed in the collinear-surround condition in Expt. 5, where the context had the same orientation as the central target. However this account cannot easily explain a similar benefit from an orthogonally-oriented context in Experiments 1–4 (and the orthogonal-surround condition in Expt. 5). Note also that neither this nor any of the above attentional accounts explain the striking objective differences observed with moving versus static stimuli without additional assumptions.

One further account based on feature-uncertainty would assume that the target and overlay mask are represented by independent mechanisms in early vision, but that the observer is uncertain which of these they should base their decisions on when asked to detect a threshold increment in contrast. When this uncertainty is resolved, sensitivity to the relevant signal improves because responses from irrelevant mechanisms can be selectively discounted from the perceptual decision [32], [35]. In the present case, a collinear context might help to identify the central target component as relevant, while an orthogonal context might help to reject the irrelevant overlay mask as irrelevant. This account can thus explain improvement of sensitivity for targets with either collinear or orthogonal surround. Furthermore, models that consider the influence of uncertainty on contrast discrimination predict a vertical shift of the TvC [22], [25], similar to that observed in Experiment 4 which we attributed to a change in the response gain parameter *k* of the transducer function. However, three points weigh against this decision-level account. Firstly, there was no objective uncertainty regarding the relevant features of the test stimulus as the test was always horizontal in Experiments 2–5, with fixed spatial frequency, and observers had ample opportunity to attune to this in baseline trials without an overlay mask. Secondly, additional assumptions are required to explain why uncertainty should be greater with drifting stimuli than with static when there is no surrounding context, and indeed why such uncertainty should correlate with the subjective appearance of plaid versus component motion as observed in Experiment 1. Finally, a similar past study of cross-orientation surround masking found no evidence for the influence of uncertainty on central contrast discrimination [19]. Thus it is more likely that the elevated thresholds in the small-mask condition arise from the difficulty in accessing the horizontal test when it coheres with the mask to form a drifting plaid, and not because of an increase in uncertainty over test orientation.

Many past studies have investigated how grating contrast sensitivity depends on the stimulation in its immediate and surrounding context [1]–[3], [13]–[16]. However only a few studies have compared superimposed orthogonal masks varying in area and overlap with target [15], [16]. Meanwhile, ‘plaid’ stimuli have typically been tested only with motion [10], though with exceptions [36]. This is therefore (to our knowledge) the first study to have measured contrast discrimination for a plaid, while independently manipulating both stimulus area and motion together. Could past contrast masking results nevertheless be generalized to account for the present results with moving and static plaids? In cross-orientation masking (i.e. ‘orthogonal’ as termed here), the weak effect of mask area [1], [5], [16], [26], [27] concurs with our findings with static stimuli, but conflicts with the strong facilitatory effect for large masks observed with moving stimuli. Past reports of cross-oriented facilitation with static annular surrounds [19], [29] might seem to explain the unmasking that we observe with a large mask. However, we do not obtain cross-oriented facilitation with the static version of the large mask. Furthermore, such facilitation cannot explain the unmasking we observed when the surround was collinear with the target (Experiment 5).

Effects of possible segmentation cues on visual discrimination have been reported before [12], [37]–[40]. For example, the masking effect of a surround on a central test can be relieved either by an orientation or contrast polarity difference between test and surround [38], or by a spatial discontinuity between these components [3], [5]–[7]. Furthermore, speed discrimination for a grating target in the context of a plaid improves when segmentation is induced via differences in contrast between drifting components [12]. However, no previous study has specifically examined effects of segmentation cues on contrast sensitivity for components of a plaid. The present study is thus the first to show that this depends on cues determining whether the plaid components bind together or peel apart. In further contrast with past studies, we avoided potential artefacts from sharp discontinuities between centre and surround (in Experiments 1 to 4), or differing contrast between individual components, by manipulating only stimulus area and motion.

The results of contrast masking studies have inspired models that account for the results in terms of excitatory and inhibitory interactions between local spatial filters in early visual cortex [1], [16]. More recent models can also account for interactions from both overlapping and annular cross-oriented components [16], or from widely-spaced flanking patches [41]. Some of these findings may be explained by interactions between mechanisms within a single visual area such as V1 [38]. Our TvC data and the fits of a contrast gain control model identify the primary effect of the surround manipulation as a modulation of the gain of the contrast response function. However, the above models (including the one used here) still require an explicit mechanistic explanation for how the contextual manipulation could influence contrast sensitivity in the manner observed, and the empirical interaction of stimulus area with motion.

Our findings implicate sources of influence from higher areas sensitive to scene organisation that can modulate the interactions between local spatial analyzers in early vision. For example, it has been proposed that feedback from higher-level mechanisms may gate or modulate the pooling of signals across orientation channels for gain control [6], [7], or lateral contextual interactions [42], [43]. Such ‘scene-organisation’ functions may be performed as early as V2, given its role in long-range grouping, and integration/segmentation of overlapping or occluding surfaces [44], [45]. Given the evident importance of motion in our study, it is also likely that some feedback comes from motion sensitive areas such as MT [46]. While it is known that end-stopped cells in V1 can use 2D features (such as contour terminations) to resolve the aperture problem, it is still unclear whether such cells can use the 2D information present in the ‘blobs’ formed by plaid intersections to compute the compound motion trajectory [47]. It is also unclear what intrinsic V1 mechanisms might effectively un-do the influence of such 2D cues on the trajectory computation, as we have observed in the context of motion with a surround. However, our findings potentially implicate mechanisms sensitive to 2D motion present as early as V1, and/or closely coupled interactions between V1, V2 and MT, as described in recent models [48], [49].

Finally, our results provide further insight into the link between two different levels of description in understanding vision: firstly the fundamental level of sensory encoding, where masking phenomena are assumed to be the result of interactions between early cortical mechanisms; and secondly the higher level of scene interpretation, which may relate more closely to our perceptual experience of distinct objects and surfaces. Though it is intuitive to suppose that encoding strictly precedes interpretation, the present results reinforce evidence for a reverse direction of influence: depending on cues for scene-interpretation, masking is enhanced when image components stick together, and relieved when the components peel apart.

## Materials and Methods

### Ethics Statement

Human observers were tested after obtaining written informed consent, following procedures approved by the Institutional Review Board at Smith-Kettlewell.

### Observers

The two authors and a total of 10 naïve observers participated for payment. Some were more practiced in psychophysical tasks than others. The authors participated in all experiments. Other observers participated in multiple experiments.

### Stimuli

For the first four experiments, stimulus presentation was controlled by a Power Mac G4/450. An 8-bit NVidia GeForce2 MX graphics card provided virtual 9-bit gray-level resolution using dithering of alternate columns of pixels with background luminance. The display was a 19″ Hitachi RasterOps Mc 7515 CRT. Displays were viewed in a darkened room from a distance of approximately 100 cm. Software was custom-written in C using the Open GL graphics library. Experiment 5 used a 10-bit ATI Radeon 7200 graphics card with a 19″ Sony G400 CRT and the Matlab Psychophysics Toolbox [50]. In all experiments, video mode was 1152×870 pixels with 75 Hz refresh rate, and background luminance was 40 cdm^−2^, with display output linearized via look-up-table.

Stimuli were presented foveally in the centre of the screen, which was marked by a white cross subtending a visual angle 0.43 degrees during inter-trial intervals. Stimuli were composed of two orthogonally-oriented gray sinusoidal gratings, at 30% Michelson contrast (except in Expt. 4, where only the mask had a fixed contrast of 30%). One was designated the ‘Test’ and the other the ‘Mask’, and both were additively superimposed on each other to produce a plaid. The Test stimulus was horizontal and the Mask was vertical, except for Experiment 1 where the Test and Mask were presented in 1 of 4 orientations: horizontal, vertical and 45 deg clockwise and counter-clockwise from vertical. Spatial frequency was 2.5 cycles per degree and drift temporal frequency was 3.2 cycles per second (or zero in Experiment 2). In the first four experiments, the test grating was presented in a two-dimensional Gaussian envelope of Standard Deviation 0.4 deg (Fig. 1a). This was always presented with a superimposed mask, which was either the same size as the test (‘Small’) and windowed by a similar Gaussian envelope (Fig. 1b) or twice the size (‘Large’), windowed by a sharp-edged circular envelope with a diameter of 2.6 deg and a flat contrast profile (Fig. 1c).

Experiments 2 to 5 had an additional ‘Baseline’ condition where the Test stimulus was presented without a mask. In these experiments also, the Test stimulus had a ‘Pedestal’ contrast with an increment added to one of the two intervals, selected randomly. The Pedestal contrast was fixed at 30%, except in Experiment 4 where it was systematically varied between 0% and 30% contrast.

In Experiment 5, a circular spatial envelope with sharp boundaries was applied to the Small Mask and the Pedestal (as well as the Large Mask as in the previous experiments), which had a diameter of 1.3deg and fixed contrast of 30%. However, the Test contrast increment had a Gaussian profile within the boundary defined by the Pedestal, but with contrast set to zero beyond this boundary. The use of sharp boundaries for the central Pedestal and Small Mask stimuli was intended to reduce spatial uncertainty for the location of the Test stimulus, which might otherwise blend smoothly into the Collinear Surround. To further demarcate the Test area, four 30° segments of a black outlined circle of 1.3deg diameter were presented outlining the boundaries of the target area. The fixation point was now a small white dot, visible only in inter-trial intervals.

Experiment 5 replicated the No Mask baseline (Figure 5a), Small Mask (Fig. 5b) and Large Mask conditions (the latter referred to here as ‘Orthogonal Surround’, Fig. 5c), but added a further ‘Collinear Surround’ condition (Fig. 5d). In this condition, the diameter of the central Pedestal stimulus was extended to 2.6deg while the orthogonal Mask had the same diameter as the small mask (1.3deg), effectively rotating the original orthogonal surround by 90° so that it had the same orientation and phase as the central Test.

### Design and Procedure

In Experiment 1, observers were asked to judge the motion direction of the central region of the image after a single presentation of drifting test and mask. Small and Large Mask conditions were tested in separate randomly interleaved blocks of 50 trials. Each observer ran a minimum of three blocks for each condition. Each block began with a fixation display. After 350 ms, the fixation cross was immediately replaced by the stimulus, which remained visible for 213 ms. The screen then stayed blank until a response from the observer, which initiated the next trial. Each trial had a test grating presented at one of 4 orientations ranging from 0° to 180°, in steps of 45°. Each test orientation had two opposite directions of drift, yielding a total of 8 drift directions. Superimposed Mask grating orientations were always orthogonal to the test orientation, and drifted in a direction 90° clockwise from the test drift direction. Observers were instructed to indicate the perceived direction of drift of the central region of the display, i.e. the region with the test grating. Perceived direction responses were entered via the numeric keypad of a standard keyboard (all numbers excluding 5), where keys ‘2’ ‘4’ ‘6’ and ‘8’ corresponded to upwards, rightwards, rightwards and downwards, respectively, while ‘1’ ‘3’ 7’ and ‘9’ corresponded to oblique directions in between.

In Experiments 2 and 3, the three conditions, Baseline, Small Mask and Large Mask were run in separate blocks of 50 trials avoiding contiguous repetition of the same condition. A Method of Constant Stimuli (MCS) was used sampling five values of increment contrast. The range of contrast increments was optimised in practice blocks to elicit near chance to near ceiling performance at minimum and maximum levels respectively. Observers performed a two-interval forced choice contrast discrimination task. Each block began with a fixation display, and a keypress initiated the trial sequence. After the fixation display that lasted 1000 ms duration, there were two stimulus intervals of 150 ms duration, separated by a blank display of 350 ms. The contrast increment was present in only one of these intervals, randomly selected. The fixation display then reappeared until a response from the observer initiated the next trial. Observers were instructed to press one of two keys on the standard keyboard, to indicate whether the increment was seen in the first or second interval (i.e. in which interval the contrast of the Test stimulus appeared higher). The threshold estimate for each condition was based on 4 blocks. In Experiment 4, similar procedures were used but we ran a total of 3 blocks (150 data points) for each pedestal contrast and masking condition.

In Experiment 5, contrast was updated on each trial using a procedure based on the QUEST algorithm. The first nine trials of each block were used to estimate an appropriate contrast increment: the same value of contrast was presented for sets of 3 trials and the adaptive procedure updated contrast based on the most frequent response in the triplet. After the first 9 trials, contrasts were adjusted depending on the response to the previous trial. This procedure was intended to help avoid gross misestimations of threshold caused by erroneous responses early in the trial. Each block contained 126 trials, and a minimum of three blocks were run for each experimental condition. The authors plus two new observers were also tested with MCS, using five values of increment contrast that bracketed threshold, with 20 trials per contrast.

## Supporting Information

Audio/Video S1 Animated demonstration of Small Mask stimulus
(0.05 MB AVI)

Audio/Video S2 Animated demonstration of Large Mask stimulus
(0.07 MB AVI)
